# Evaluating the Effectiveness of Epidural Steroid Injections in Relieving Pain in a Single-Center Retrospective Cohort

**DOI:** 10.7759/cureus.76916

**Published:** 2025-01-04

**Authors:** Annabel Kim, Joshua G Sanchez, Marc A Abdou, Zorica Buser, David Cheng, Trevor Pickering, Gene Tekmyster

**Affiliations:** 1 Orthopaedic Surgery, University of Southern California Keck School of Medicine, Los Angeles, USA; 2 Orthopaedic Surgery, Yale School of Medicine, New Haven, USA; 3 Orthopaedic Surgery, New York University Grossman School of Medicine, New York, USA; 4 Orthopaedics and Rehabilitation, Veteran Affairs Long Beach Healthcare System, Long Beach, USA; 5 Public Health Sciences, University of Southern California, Los Angeles, USA

**Keywords:** conservative treatment, conversion to surgery, epidural steroid injections, esi, lumbar spine fusion surgery, non-surgical intervention, nrs, numerical rating scale for pain, radiculopathy

## Abstract

Background

Epidural steroid injections (ESIs) are a common conservative treatment for mitigating radicular pain and are often used to relieve pain, increase function, and improve mobility. However, their efficacy and duration of pain relief are relatively unclear because of the variability in clinical indications, injection techniques, injection mixtures, the number of allowable injections, and the lack of standard and objective outcome measures in the literature.

Objectives

This study aimed to characterize the effectiveness of ESIs in improving pain, measured with numerical rating scale (NRS) scores, and their relationship with subsequent lumbar spine surgery within a one-year period.

Methods

Patients who received a lumbar ESI from January 2018 to March 2022 in the Keck Medical Center of the University of Southern California were identified. Only patients with a one-year follow-up and no traumatic injuries were included. Exclusion criteria included a prior lumbar ESI within five years prior to January 2018. Demographics, comorbidities, injection information, and NRS scores were extracted. NRS score comparisons were analyzed with the Wilcoxon signed-rank test. Significance was defined at p ≤ 0.05.

Results

A total of 143 ESI patients were identified. The patient population consisted of 62 (43.36%) male, 81 (56.64%) female, and a median age of 63 years (IQR: 51,73). Patients who were one- and five-months post-ESI had the greatest median change in NRS of -3 (IQR: -7,0) (p < 0.05 for all). At one year post-ESI, there was a median decrease in NRS scores by 2 (IQR: 0,5). Of the cohort, only 28 (27.20%) patients went on to have lumbar spine surgery within a year.

Conclusion

The data suggests ESIs may be effective at relieving pain for at least one year. The data provides some evidence that ESIs are most reliable at relieving pain up to the five-month mark, after which their efficacy decreases.

## Introduction

Low back pain (LBP) and associated radicular syndromes are significant health and socioeconomic issues that account for over 100 billion dollars spent annually on care in the United States [1]. Radicular pain is believed to stem from spinal stenosis, causing inflammation at the junction between the nerve root and narrowed tissue [2]. Existing literature suggests that treatment of acute low back pain (less than six-week pain duration) has more favorable outcomes compared to chronic low back pain (more than three-month pain duration) and patients with more acute pain are more likely to recover overall [3]. Therefore, researching the efficacy of early interventions in treating low back pain is crucial to delaying the progression of symptoms and associated functional limitations.

Epidural steroid injections (ESIs) are a common conservative treatment for relieving radicular pain from associated lumbar spine etiologies [2, 4, 5]. They are the most common procedural treatment of low back pain and related radicular syndromes in the United States, with utilization rates more than doubling within the past decade [4]. Lumbar epidural injections can be performed via interlaminar, transforaminal, and caudal approaches injecting steroids, local anesthetics, or a combination of both. These injections are utilized with the goal of relieving pain, increasing function, and improving mobility, which may delay further progression of symptoms. With the increasing prevalence of low back pain [1], understanding pain improvement and the potential delay role of ESIs and rates of subsequent surgical treatment following treatment is vital. 

Even so, the efficacy and duration of pain relief from ESIs remain relatively unclear in the current literature due to the variability in clinical indications, injection techniques, injection mixtures, the number of allowable injections, and the lack of standard and objective outcome measures [4-6]. A prior meta-analysis by Bicket et al. demonstrated mixed results on whether ESIs mitigate the need for future spine surgery, with low- to moderate-quality evidence that ESIs delay surgical treatment only in the short term (< 1 year) [4]. Similarly, a systematic review by Vorobeychik et al. in 2016 showed that non-image-guided lumbar ESIs appeared to relieve pain only in the first three to six weeks [7]. However, another systematic review of placebo-controlled trials of ESIs found no significant improvement in pain relief at either three to six months [8].

With the lack of clarity regarding ESI efficacy in relieving pain and delaying surgical treatment, the aims of the present study were to evaluate pain relief through numerical rating scale (NRS) pain scores as well as the rate of subsequent lumbar spine surgery within one year of the index ESI in a retrospective, single-center study design. We hypothesized ESIs would relieve pain in the short-term (< 6 months) and delay surgery within the one-year follow-up period.

## Materials and methods

Study population

This is a retrospective observational study of 143 patients who received transforaminal lumbar ESIs from three interventional physiatrists in the Keck Medical Center of the University of Southern California from January 2018 to March 2022. The injections consisted of a mixture of dexamethasone, lidocaine, and normal saline. Exclusion criteria included a prior lumbar ESI within five years to January 2018, back pain etiology resulting from traumatic injury, and a lack of available data within a year following the index ESI. All included patients also had a primary diagnosis of lumbar radiculopathy (identified through the International Classification of Diseases, Tenth Revision (ICD-10) code M54.16).

The study was conducted in accordance with the Declaration of Helsinki and was approved on 03/19/2021 by our institutional review board (IRB), the University of Southern California Human Research Protection Program under HS-20-01035. Due to the aggregated and de-identified nature of the data obtained in the retrospective study design of the current study, IRB exemption from informed consent was approved. All data were extracted from the electronic medical record and any protected health information (including names, screening IDs, medical record numbers, and mobile phone numbers) was de-identified and not recorded.

Demographics

Collected demographic variables included patient age, gender, body mass index, smoking status, and concomitant comorbidities (hypertension, diabetes mellitus, hyperlipidemia, pulmonary conditions, cardiovascular conditions, renal conditions, and/or mental illnesses such as anxiety or depression).

NRS pain scores

NRS pain scores, which grade pain from a scale of 0-10 in which 10 is considered to be the worst imaginable pain, were collected [9]. These scores were compiled up to a month prior to the index injection and 1 month, 2 months, 3 months, 4 months, 5 months, 6 months, 9 months, and 1 year following the index injection. 

NRS scores were then categorized based on the length of pain the patient experienced before the index injection, with categories defined as ≤ 3 months, 3 < x ≤ 6 months, 6 months < x ≤ 1 year, and > 1 year.

Finally, patients with lumbar spine surgery within a year of the index ESI were identified by examining patient intake forms and clinic notes during a patient’s one-year follow-up appointment. NRS scores were then categorized based on whether subsequent lumbar spine surgery within one year of the index injection was performed.

Statistical methods

Because of the study’s non-normal data, medians and interquartile ranges (IQR) were used to describe the data. For the analysis of NRS scores, the Wilcoxon signed-rank test was used to evaluate whether patients reported a difference in the median NRS score at each specified time point post-injection in comparison to the baseline reported pain. This method was utilized for all NRS score comparisons. The Wilcoxon signed-rank test was utilized as it is a non-parametric statistical test that may be used on non-normal data such as NRS scores.

All statistical tests were completed with R version 4.3.2 (R Foundation for Statistical Computing, Vienna, Austria) and Microsoft Excel (Microsoft Corp., Redmond, WA). Statistical significance was defined as α ≤ 0.05 for all tests. P-values were adjusted for the false discovery rate. 

## Results

Study population

A total of 143 patients were identified over a 12-month period. For these 143 patients, the median age was 63 (IQR: 51, 73) and the median BMI was 26.65 (IQR: 23.44, 30.10). The total patient sample consisted of 62 males (43.36%) and 81 females (56.64%). The most prevalent comorbidities (Table 1) included hypertension (n=65; 45.45%), hyperlipidemia (n=39; 27.27%), and some form of mental health diagnosis (n=30; 20.98%). The majority of patients (n=108; 75.52%) reported that they had never used nicotine prior to their ESI treatment. However, 30 (20.98%) reported to have smoked in the past, and 5 (3.50%) patients reported currently smoking. Of the total injections, 25 (17.86%) were 16 mg of dexamethasone and 1.5 cc of 1% lidocaine; 36 (25.40%) were 16 mg of dexamethasone, 1.5 cc of 1% lidocaine, and 1 cc of normal saline; 64 (44.44%) were 10 mg of dexamethasone and 2 cc of 1% lidocaine; 1 (0.40%) was 8 mg Kenalog and 2 cc of normal saline; 2 (1.19%) 80 depo-medrol and 2 cc 1% lidocaine; and 15 (10.71%) were 15 mg of dexamethasone and 1.5 cc of 1% lidocaine. All patients received transforaminal ESIs with varying mixtures.

**Table 1:** Demographics and comorbidities of the overall patient population. ^*^ Median (IQR); all other values are represented as n (%). IQR: interquartile range, n: number

Characteristic	Values
Age^*^	63 (51, 73)
Gender	
Male	62 (43.36%)
Female	81 (56.64%)
Hypertension	65 (45.45%)
Diabetes	18 (12.59%)
Hyperlipidemia	39 (27.27%)
Pulmonary diagnoses	13 (9.09%)
Cardiac diagnoses	26 (18.18%)
Renal diagnoses	16 (11.19%)
Mental health diagnoses	30 (20.98%)
BMI^*^	26.65 (23.44, 30.10)
Smoking status	
Past	30 (20.98%)
Current	5 (3.50%)
Never	108 (75.52%)

NRS score changes for the total study cohort

**Figure 1:**
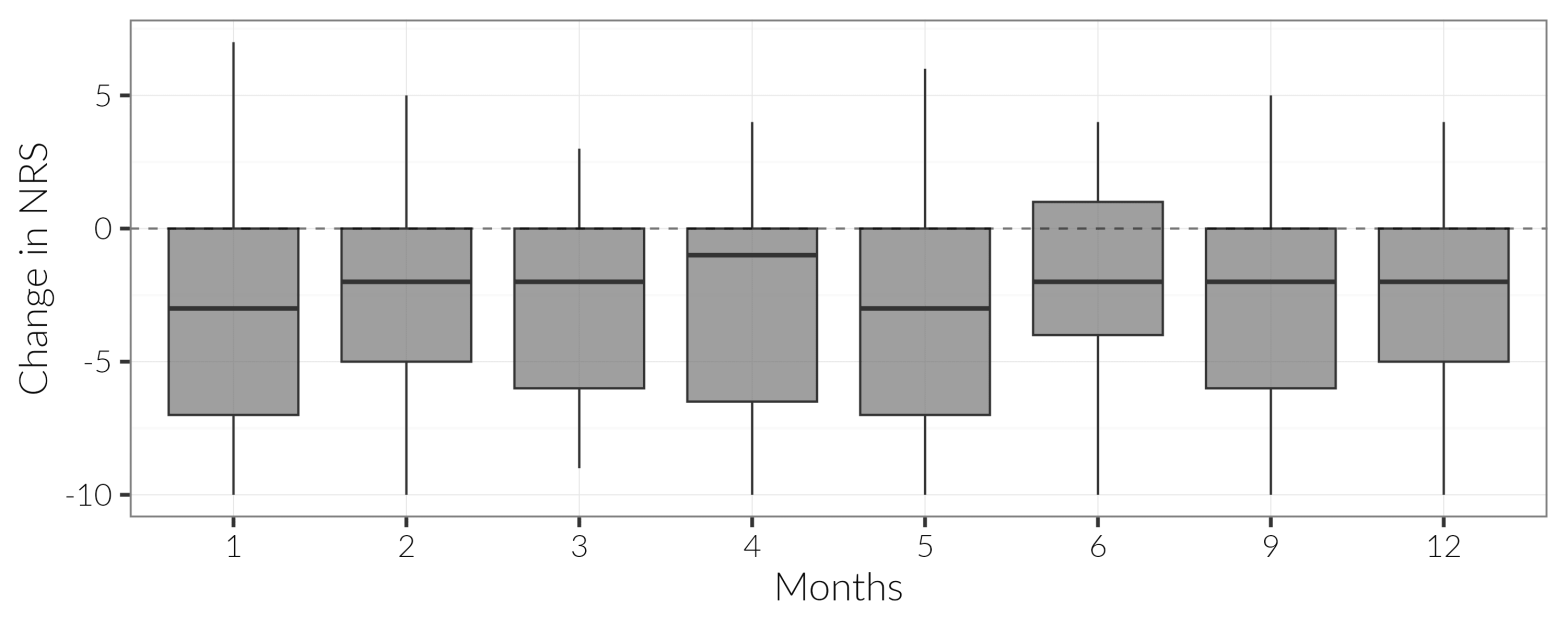
Boxplot depicting the change in NRS scores from baseline, categorized by month, across the 12-month postoperative period. NRS scores for all months compared to baseline were significantly different (p<0.001). These scores were compiled up to a month prior to index injection and 1 month, 2 months, 3 months, 4 months, 5 months, 6 months, 9 months, and 1 year following the index injection. NRS scores were then categorized based on the length of pain the patient experienced before the index injection, with categories defined as ≤ 3 months, 3 < x ≤ 6 months, 6 months < x ≤ 1 year, and > 1 year. NRS: numerical rating scale

Overall, NRS pain scores decreased one month after the first study injection and stayed consistently low throughout the year. A statistically significant decrease in NRS scores was found throughout the year. The one-month and five-month post-ESI time points displayed the greatest median change in NRS of -3 (IQR: -7,0). One year following the ESI, the median change in NRS score was -2 (IQR: -5,0) (Figure 1, p < 0.05 for all). 

NRS score changes based on the length of pain

**Figure 2:**
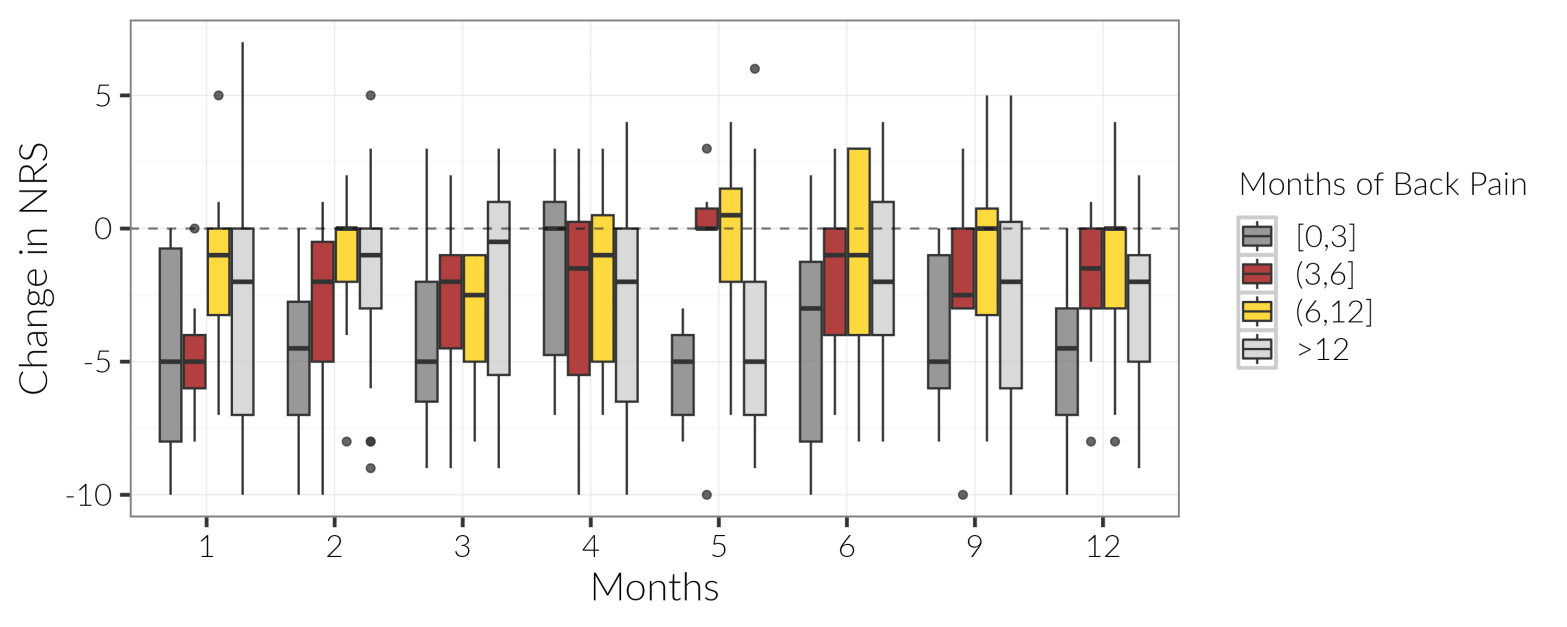
Boxplot of NRS change scores from baseline by month and pain group. These scores were compiled up to a month prior to index injection and 1 month, 2 months, 3 months, 4 months, 5 months, 6 months, 9 months, and 1 year following the index injection. NRS scores were then categorized based on the length of pain the patient experienced before the index injection, with categories defined as ≤ 3 months, 3 < x ≤ 6 months, 6 months < x ≤ 1 year, and > 1 year. NRS: numerical rating scale

Of the study population, 28 (19.58%) reported a history of up to three months of low back pain before their index ESI, 18 (12.58%) patients indicated pain for greater than three months and up to six months, 23 (16.08%) indicated pain greater than six months up to a year, and 68 (47.55%) reported pain lasting over a year. The pain length experienced pre-injection was not found for six (4.19%). Although not statistically significant, it was noted that patients with shorter back pain histories experienced greater pain relief following ESIs (Figure 2).

Conversion to surgery

**Figure 3:**
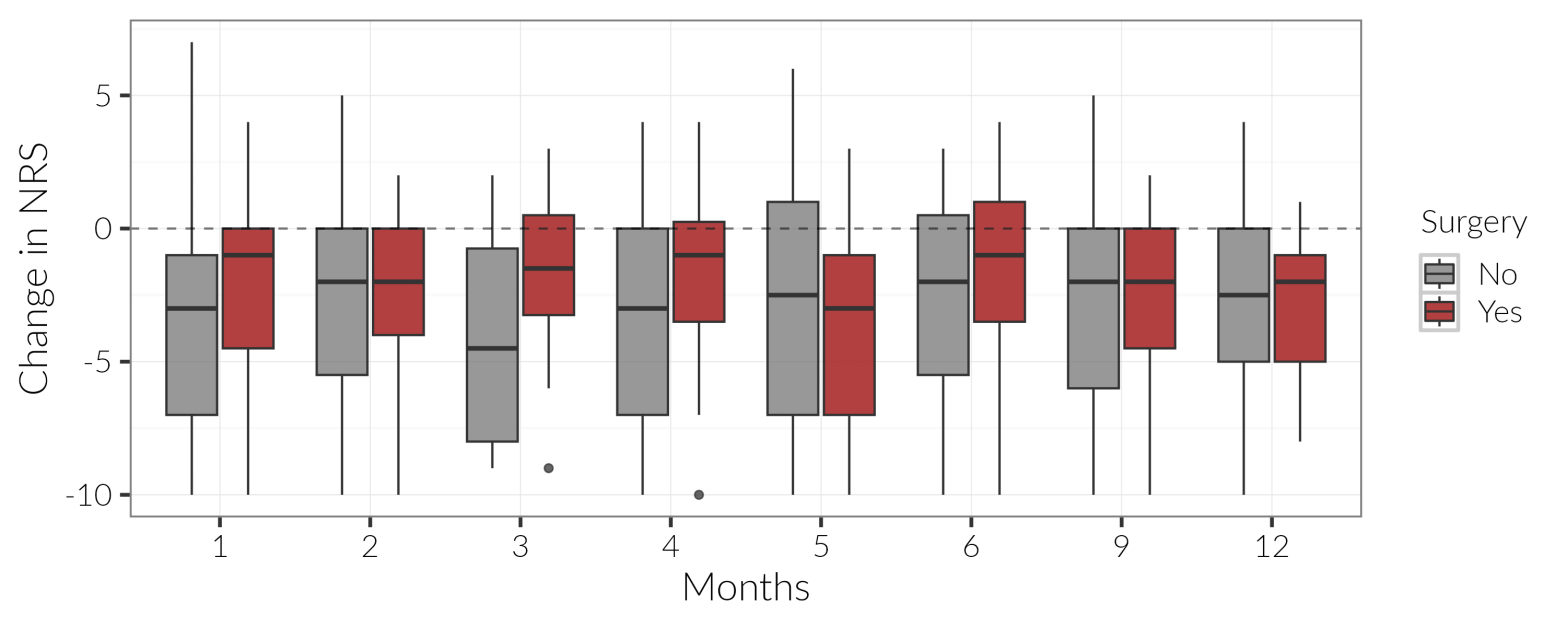
Boxplot of NRS change scores from baseline by month and surgery status. These scores were compiled up to a month prior to index injection and 1 month, 2 months, 3 months, 4 months, 5 months, 6 months, 9 months, and 1 year following the index injection. NRS scores were then categorized based on the length of pain the patient experienced before the index injection, with categories defined as ≤ 3 months, 3 < x ≤ 6 months, 6 months < x ≤ 1 year, and > 1 year. NRS: numerical rating scale

A total of 34 (23.78%) patients who underwent lumbar spine surgery within one year of the index injection were identified. Of these 34 patients, 11 (35.00)% of patients reported over a year of pain, and nine (29.00%) reported up to three months of pain. From this cohort, five (15.00%) patients who went on to have surgery received one ESI, 10 (29.80%) received two, and 10 (30.80%) received at least three ESIs prior to surgery. The surgery conversion rate doubled from those who received one to two injections but leveled off for those who received three. However, it is important to note the overwhelming majority of patients (n=109; 76.22%) did not proceed to have spine surgery. At most time points post-injection, patients in the surgical group appear to have reported less pain relief from the ESI (Figure 3). However, this difference in NRS scores by surgery status was not statistically significant. 

## Discussion

Low back pain is a prevalent health problem not only in the United States but the rest of the world, affecting most individuals at least once in their lives, with only the common cold surpassing the frequency of complaints heard by primary care physicians [1]. Hoy et al. assert that low back pain is most prevalent in women and individuals aged 40 to 80 [9]. The higher frequency of severe low back pain in older patients marks a rising problem in the context of an increasingly aging global population [9]. ESIs are one of the most common procedures used to treat low back pain, particularly syndromes with concomitant radiculopathy [8]. The corticosteroids in these injections are thought to alleviate pain by reducing inflammation and blocking nociceptive nerves [10]. However, the effectiveness of this procedure and length of pain relief are currently inconclusive with varying literature findings. In light of this lack of clarity, the present study aimed to evaluate pain relief and rate of conversion to lumbar spine surgery within one year of the injection in a retrospective single-center study. 

The study population supports the existing literature that more women and older individuals are afflicted with low back pain [11, 12]. Additionally, the findings of this study describe a statistically significant reduction of NRS scores in patients who received ESIs over a 12-month period. Patients reported the most amount of pain relief around one month after their epidural steroid injection. Furthermore, patients reported relief for up to five months with decreasing pain relief reported for up to one year. This is consistent with a prior systematic review study by Manchikanti et al. and a double-blind randomized controlled trial by Friedly et al. that demonstrated if ESIs led to pain relief for six weeks post-injection, these effects maintained efficacy up to 12 months [8, 13, 14].

Furthermore, the results of this study present the transient effects of ESIs in relieving low back pain and radicular pain which is in line with prior literature [2, 4, 15]. Radcliff et al. hypothesized that transitory pain relief effects of ESIs may be due to the treatment only masking pain stimuli rather than healing the source of pain [15]. Additionally, the diminishing effects of ESIs may result from the progression of disease following the injection [4]. Taken together, these data suggest additional randomized controlled trials (RCTs) should be done to further study the potentially transient nature of ESIs.

The analysis of pain history length prior to the first ESI showed the majority of study patients reported either 0-3 months or >12 months pain histories, suggesting ESIs may be preferentially considered for those in the extremes of pain duration. Although not statistically significant, the study’s data prompts consideration that ESIs may better relieve pain for patients who have experienced shorter histories of low back pain as patients with up to three months of pain trended greater improvement. The early utilization of ESIs may allow for a more impactful anti-inflammatory effect that could prevent epidural and perineural fibrosis, potentially averting further damage to the spine and worsening symptoms [16].

Furthermore, patients who had conversions to lumbar spine surgery reported less pain relief from their ESI though statistical significance was not reached. It is worth noting that the surgical conversion rate doubles in patients with two ESIs versus one, but levels off at three ESIs. However, there is no statistically significant difference in the surgical conversion rate based on the number of injections received (p = 0.11). Although not significant, patients who have more than one ESI were found to be more likely to undergo lumbar surgery within one year of their last ESI. These findings are supported by prior research from Friedly et al. that concluded patients with multiple ESIs were more likely to undergo lumbar surgery within six months after their last ESI [16]. 

Several limitations should be considered for this study. As this was a retrospective study, patients could have been concurrently using other conservative treatments, such as acupuncture, exercise therapy, etc. Additionally, as not all patients may have been surgical candidates, this may have confounded the results of our analysis of pain relief based on subsequent lumbar spine surgery. Further, while this study may not be generalizable to the entire ESI patient population as the patients included in this study were treated by three interventional spine physiatrists at a single academic center, it should be noted that in a well-selected and appropriately indicated population, epidural corticosteroid injections are a valid and effective treatment modality. Furthermore, the present study focused on the analysis of patients with lumbar radiculopathy and no other spine pathologies. As such, we could not account for the effects of various spinal pathologies on the analysis such as central spinal stenosis. Lastly, the largest limiting factor to the current study was the lack of significant statistical power. Nevertheless, the results of this study support prior research regarding the efficacy of ESIs in low back pain relief. Further, the current study may prompt valuable prospective research to characterize what patient populations ESIs work best to help mitigate subsequent lumbar spine surgery. 

## Conclusions

The results of this study suggest that ESIs may relieve pain for up to one year. Specifically, patients reported the greatest pain relief at one month, persisting up to the five-month mark and tapering off by the one-year point. Although not reaching statistical significance, the present study demonstrated a trend in which ESI patients who had conversions to lumbar spine surgery reported a longer pain history and less pain relief from their injection. Additional prospective research may further clarify the effectiveness of ESIs in mitigating conversion to lumbar spine surgery. Overall, this study adds to the body of literature supporting the use of ESIs in the appropriately indicated population.
